# Antibiogram of bacteria isolated from automated teller machines in Hamadan, West Iran

**DOI:** 10.3205/dgkh000288

**Published:** 2017-02-02

**Authors:** Hassan Mahmoudi, Mohammad Reza Arabestani, Mohammad Yousef Alikhani, Iraj Sedighi, Hamed Farhadi Kohan, Mohammad Molavi

**Affiliations:** 1 Microbiology Department, Hamadan University of Medical Sciences, Hamadan, Iran; 2 Brucellosis Research Center, Hamadan University of Medical Sciences, Hamadan, Iran; 3 Pediatric Departments, Hamadan University of Medical Sciences, Hamadan, Iran

**Keywords:** automated teller machines, bacteria, keyboard, antibiotic pattern

## Abstract

**Aim:** Bacteria are ubiquitous in the environment. In keeping with the continued expansion of urbanization and the growing population, an increasing number of people use automated banking, i.e. automated teller machines (ATMs). The aim of this study was to investigate the bacterial contamination and its antibiotic sensitivity on computer keyboards located at ATMs in Hamadan province, Iran.

**Method:** Out of 360 ATMs at four locations in Hamadan, 96 were randomly selected for this study. The antibiotic susceptibility pattern of all isolates was determined by the agar disk diffusion method using gentamicin (10 µg), vancomycin (30 µg), trimethoprim/sulfamethoxazole (25 µg), amikacin (30 µg), tobramycin (10 µg), cephalotin (30 µg), norfloxacin (5 µg), and ceftizoxim (30 µg) disks.

**Results:** Melli and Saderat Banks had the most frequently contaminated ATMS, with 18 (27.7%) and 12 (18.5%), respectively. The most frequently isolated bacteria were *Staphylococcus epidermidis* in 12 (18.5%) ATMs, *Pseudomonas aeruginosa* in 12 (18.5%), *Bacillus subtilis* in 11 (16.9%), *Escherichia coli* in 6 (9.2%), *Klebsiella *spp. in 8 (12.3%), *Enterobacter *spp. in 2 (3.1%), *Bacillus cereus* in 6 (9.2%), *Staphylococcus aureus* in 3 (4.6%), and *Micrococcaceae *spp. in 5 (7.69%) cases. All isolated bacteria were susceptible to gentamicin, cephalotin, tobramycin, amikacin, norfloxacin, and vancomycin. The *S. aureus* resistance rate to trimethoprim/sulfamethoxazole was 50%.

**Conclusion:** All tested ATM keyboards were contaminated with at least one species of bacteria. Based on these findings, it is recommendable to disinfect the hands after entering one’s own apartment, work area or a hospital, in order to hinder the spread of critical pathogens in the personal environment or in the hospital.

## Zusammenfassung

**Zielsetzung:** Bakterien sind ubiquitär in der Umwelt verbreitet. Verbunden mit der wachsenden Bevölkerung, der Urbanisation und dem Zeitmangel wächst die Inanspruchnahme von Geldautomaten (ATM). Daher sollten die bakterielle Kontamination und deren Antibiotikaresistenz auf Tastaturen von ATM in der Provinz Hamadan, Iran, untersucht werden.

**Methode:** Von 360 ATM wurden 96 randomisiert ausgewählt. Die Antibiotikaempfindlichkeit wurde im Agardiffusionstest unter Verwendung von Plättchen mit Gentamicin (10 µg), Vancomycin (30 µg), Trimethoprim/Sulfamethoxazol (25 µg), Amikacin (30 µg), Tobramycin (10 µg), Cephalotin (30 µg), Norfloxacin (5 µg) und Ceftizoxim (30 µg) ermittelt.

**Ergebnisse:** Die höchste Kontamination wurde bei 18 (27,7%) bzw. 12 (18,5%) der ATMs von Melli und Saderat Banken nachgewiesen. Am häufigsten wurden *Staphylococcus epidermidis* und *Pseudomonas aeruginosa* je 12-mal (18,5%), *Bacillus subtilis* 11-mal (16,9%), *Klebsiella *spp. 8-mal (12.3%), *Escherichia coli* und *Bacillus cereus* je 6-mal (9,2%), *Micrococcaceae *spp. 5-mal (7,69%), *Staphylococcus aureus* 3-mal (4,6%) und *Enterobacter *spp. 2-mal (3,1%) nachgewiesen. Alle isolierten Bakterien waren empfindlich gegen Gentamicin, Cephalotin, Tobramycin, Amikacin, Norfloxacin und Vancomycin. Der Anteil von *S. aureus* mit Resistenz gegen Trimethoprim/Sulfamethoxazol betrug 50%.

**Schlussfolgerung:** Alle untersuchten ATM Tastaturen waren mit mindestens einer Bakterienspecies kontaminiert. Daher erscheint es sinnvoll, z.B. nach dem Betreten der Wohnung, des Arbeitsbereichs oder eines Krankenhauses eine Händedesinfektion durchzuführen, um keine kritischen Pathogene in das persönliche Umfeld bzw. in das Krankenhaus einzubringen.

## Introduction

Bacteria exist everywhere in the environment and are able to persist or even grow on any surface [1], [2]. Although most bacteria are harmless, some of them are pathogenic, especially in people with weakened immune system. Due to the ongoing development and expansion of urbanization, as well as the increasing population, people do not have enough time to use traditional banking systems and have embraced new developments in electronic banking, such as ATMs (automated teller machines). Today, the extended use of electronic technologies is considered a source of bacterial contamination [3]. In general, microbes can persist or grow on many surfaces, such as those found in restaurant kitchens and hospital environments, as well as on standard office equipment such as computer keyboards, telephones, cellphones, and ATMs [3], [4], [5], [6], [7]. It was estimated that worldwide about 4.2 million ATMs have been used in 1960 [8], [9], [10]. The routine use of these devices involves inserting the card in the machine and using the fingers to enter the password [9]. Phones and devices with metal keyboards are easily contaminated with pathogenic microorganisms, and bacterial colonization and biofilm formation has been investigated by several researchers on metallic surfaces [10], [11], [12]. Today, the presence of electronic devices has increased exponentially in almost all areas of life the environment (playground equipments, ATM keyboards, kitchen sinks, office desks, computer keyboards, escalator handrails, elevator buttons and with the spread of supermarkets and hypermarkets the shopping carts handles). The US Centers for Disease Control and Prevention has estimated that there were 4.5 infections per 100 hospitalizations in 2002 [13], [14], [15]. The aim of this study was to assess the bacterial contamination of ATMs in Hamadan, western Iran.

## Materials and methods

Out of 360 ATMs at four locations in Hamadan (number provided by the central bank of the islamic republic of Iran) 96 ATMs were randomly selected. Using a sterile swab soaked in saline, samples were taken from the surfaces of the ATM keyboards. The swab was put into nutrient broth media and transferred to the microbiology laboratory of the University of Medical Sciences, Hamadan and incubated for 30 min. In order to differentiate microorganisms, swabs were cultured on blood agar and MacConkey agar plates and incubated at 37°C for 18–24 hours. Identification of the isolated bacteria was performed using standard microbiological methods [16]. For all isolated strains, antibacterial susceptibility was tested using the standard Kirby-Bauer disk agar diffusion (DAD) method on Mueller Hinton agar (Merk Co., Germany) according to the clinical and laboratory standards institute guidelines (CLSI; 2015, M100-S25) [17] using gentamicin (10 µg), vancomycin (30 µg), trimethoprim/sulfamethoxazole (25 µg), amikacin (30 µg), tobramycin (10 µg), cephalotin (30 µg), norfloxacin (5 µg), and ceftizoxim (30 µg) disks (Mast Co.UK). 

All statistical analyses were performed using the SPSS software package, version 21.

## Results

A total of 65 positive samples (67.7%) of the ATM keyboards were obtained (Table 1 (Tab. 1)).

The most frequently contaminated ATMs belonged to the Melli and Saderat banks, with 18 (27.69%) and 12 (18.46%) cases, respectively (Table 2 (Tab. 2)). The prevalence of isolated bacteria included *Escherichia coli* on 6 (9.23%) ATM keyboards, *Klebsiella *spp. on 8 (12.30%), *Enterobacter *spp. on 2 (3.07%), *Bacillus cereus* on 6 (9.23%), *Bacillus subtilis* on 11 (16.92%), *Staphylococcus epidermidis* on 12 (18.48%), *Staphylococcus aureus* on 3 (4.61%), *Micrococcaceae *spp. on 5 (7.69%) and *Pseudomonas aeruginosa* on 12 (18.64%) keyboards (Table 3 (Tab. 3)). The sensitivity patterns to tested antibiotics are shown in Table 4 (Tab. 4).

## Discussion

The findings of this study showed that ATM keyboards can be considered a source of bacterial infections, similar to other contaminated surfaces in public places, such as telephones and door handles. Because most people with different levels of hygiene and health standards use these machines, they can be widely involved in absorbing, harboring, and transferring infectious microorganisms. Contaminated hands touching an ATM keyboard can transfer pathogens to the keyboard and bills, ultimately facilitating the spread of infectious diseases [12], [13], [14], [15]. In our study, the most commonly isolated microbes were members of the *Enterobacteriaceae*, especially *E. coli* as an intestinal pathogen. The results of a study conducted by Gontijo Filho et al. [1] showed that ordinary washing hands cannot eliminate the microorganisms completely; it can only reduce contamination with microbial pathogens. In another study, isolation of Coliforms from coins, paper money, and public telephones has been reported [2]. In research performed by Karabay et al. in Turkey, 111 samples from mobile phones had contamination with microbes that can cause hospital infections. A study in which samples were collected from clothing (e.g. coats, ties) and medical equipment such as stethoscopes and clothing labels, showed microbial contamination [18], [19], [20]. A study by Neely et al. [21] in Ohio, USA on plastic and fabric demonstrated contamination with *Enterococcus *spp. and *Staphylococcus *spp. In a study by Ramesh in India [22], of 101 samples collected from mobile phones, 45% were colonized with Gram-positive and 15% with Gram-negative bacteria. Ashgar and El-Said [23] found that 64% of 96 collected swab samples from ATM slots, buttons, and door handles in Mecca city were negative and 26% were positive, a lower percentage than in our study. However, the bacterial spectrum was similar (*E. faecalis*, *Bacillus *spp. and *E. coli*). In a study on external surface of computer keyboards and computer mice, Malik et al. [24] found all samples to be contaminated with pathogenic bacteria (*E. coli*, *Salmonella*, *Shigella*, and *Staphylococcus *spp.), with *E. coli* dominating the isolates. Another paper, similar to the current study, showed that ATM surfaces were contaminated by pathogenic bacteria [25]. Furthermore, other authors obtained Gram-positive bacteria from currency notes and computer keyboards [26], and Gram-negative bacteria from hospital curtains, cell phones, hospital staff coats, and ties [26], [27]. Similar to the present study, ATMs in Ebonyi state, Nigeria, were positive for *S. aureus*, coagulase-negative *Staphylococcus*, *Streptococcus *spp., *Pseudomonas *spp.*,* *Enterobacter *spp. and *E.coli* [28]. In our study, the rate of Gram-positive bacteria was higher than that of Gram-negative bacteria. Fazeli et al. [29] isolated *P. aeruginosa* from the hospital staff, equipment and clinical samples. The isolates from the environmental samples and staff’s hands did not show significant differences in antibiotic resistance patterns [29], but the current study on *P. aeruginosa* strains isolated from the ATM keyboards showed that they were sensitive to all studied antibiotics. This discrepancy might be due to different geographical locations. The results of the present study are in accordance with those of Abban et al. [9] and Okoro Nworie et al. [30], who reported the presence of *Staphylococcus *spp., *E. coli*, and *Klebsiella* spp. on the keyboards of ATMs. All studies on ATMs demonstrate that they are a possible factor in transmitting infectious diseases.

## Conclusions

The results of this study show that some pathogens found on ATM keyboards, such as *S. aureus* and *P. aeruginosa*, express resistance to a variety of antibiotics. The ATMs are located throughout town and, especially when near hospitals, are capable of adsorbing and transmitting pathogens. It is imperative that these devices be considered as potential vehicles in the transmission of infections. Since the use of these devices is unrestricted, a health guideline is necessary to prevent infections [5]. There is no practical means of continually disinfecting such equipment. It is questionable that frequent cleaning by an operator would be enough to interrupt cross contamination, because after the next touch, contamination could be as high as before. It appears more feasible to disinfect the hands after entering one’s own apartment, work area, or a hospital, in order to stop the spread of environmentally acquired pathogens to the personal environment or the hospital. This makes sense because a prospective, controlled, intervention-control study implementing untargeted hand disinfection at work places in a public administration significantly reduced common cold, fever, coughing, and diarrhea [31].

## Notes

### Competing interests

The authors declare that they have no competing interests.

### Acknowledgements

This research was financially supported by a grant from Students Research Center, Hamadan University of Medical Sciences.

## Figures and Tables

**Table 1:**
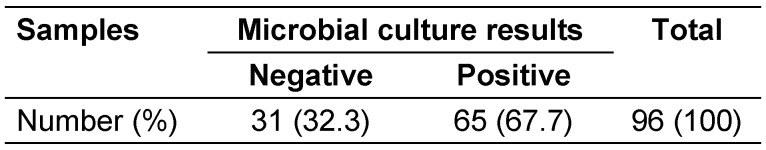
The frequency of bacterial contamination of ATMs

**Table 2:**
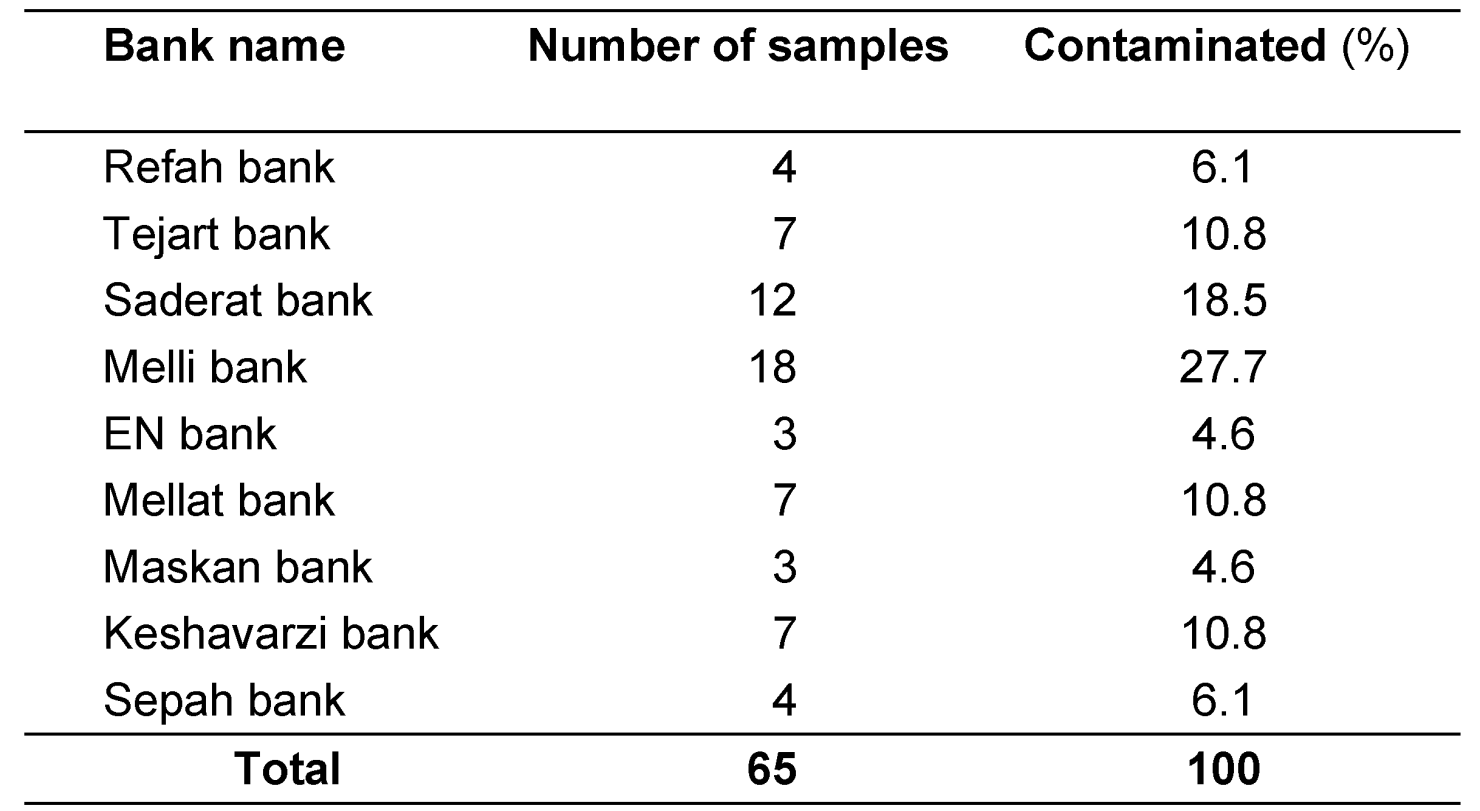
The number of contaminated ATMs according to banks

**Table 3:**
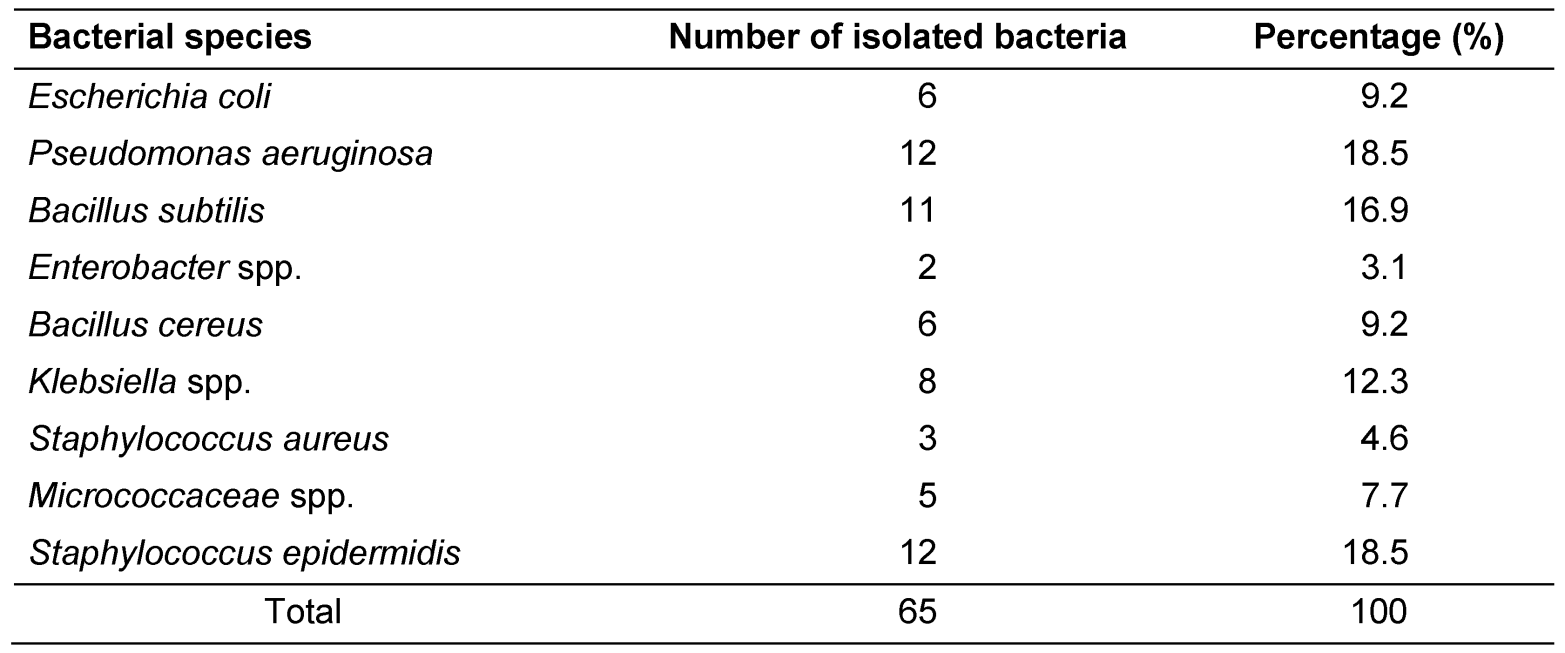
Distribution of bacterial species isolated from ATMs

**Table 4:**
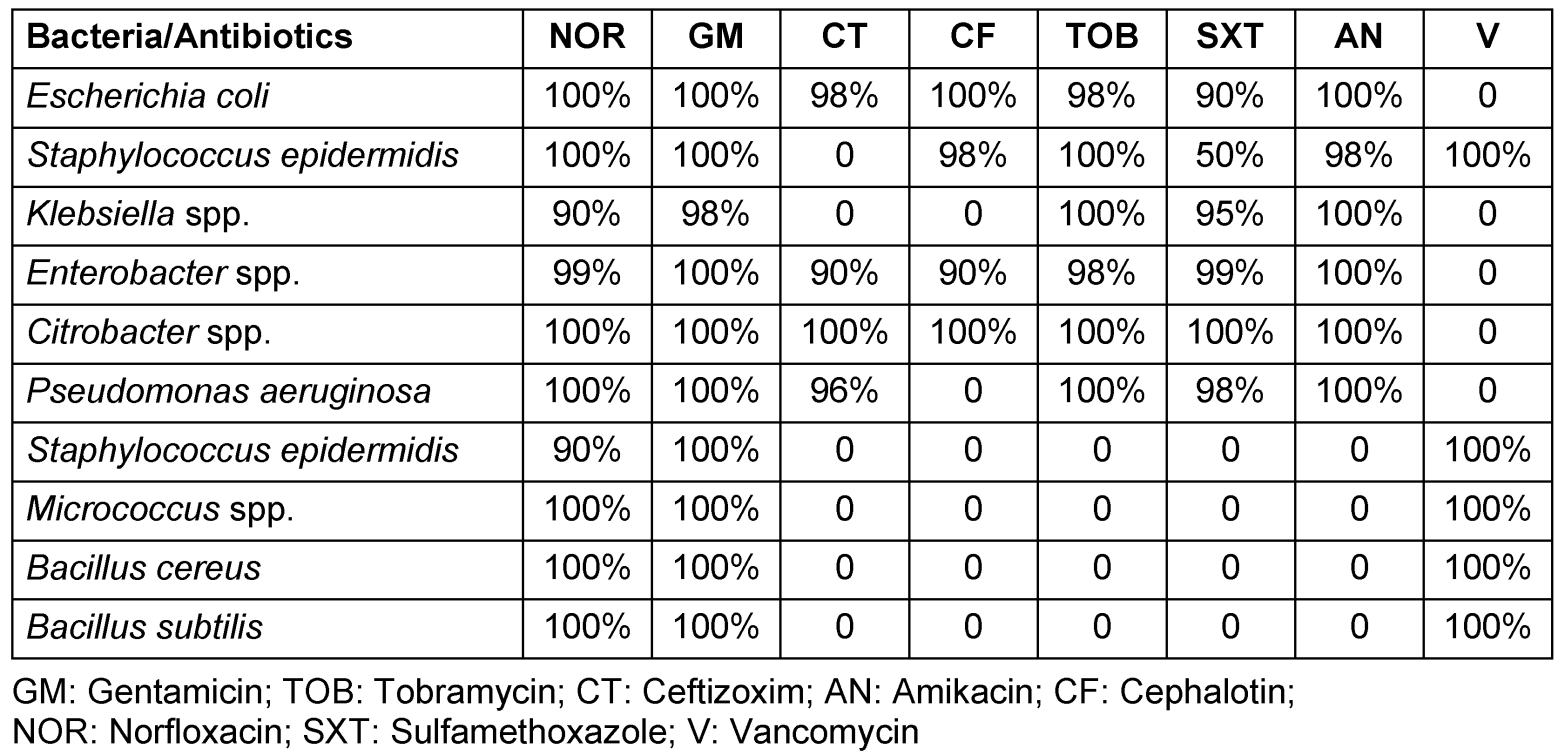
Antimicrobial susceptibility patterns of bacterial strains isolated from ATMs
